# Effectiveness of Plasmocure™ in Elimination of *Mycoplasma* Species from Contaminated Cell Cultures: A Comparative Study versus Other Antibiotics

**DOI:** 10.22074/cellj.2019.5996

**Published:** 2019-02-25

**Authors:** Vahid Molla Kazemiha, Shahram Azari, Mahdi Habibi-Anbouhi, Amir Amanzadeh, Shahin Bonakdar, Mohammad Ali Shokrgozar, Reza Mahdian

**Affiliations:** 1 National Cell Bank of Iran, Pasteur Institute of Iran, Tehran, Iran; 2 Department of Molecular Medicine, Pasteur Institute of Iran, Tehran, Iran

**Keywords:** Cell Culture, Cytotoxicity, *Mycoplasma*, Treatment

## Abstract

**Objective:**

*Mycoplasmas spp.* is among major contaminants of eukaryotic cell cultures. They cause a wide range of problems associated with cell culture in biology research centers or biotechnological companies. *Mycoplasmas* are also resistant to several antibiotics. Plasmocin™ has been used to treat cell lines but Plasmocin™-resistant strains have been reported. InvivoGen has developed a new anti-*Mycoplasma* agent called Plasmocure™ in order to eliminate resistant *Mycoplasma* contamination. The aim of this study was the selection of the best antibiotics for treatment of *mycoplasma* in cell cultures.

**Materials and Methods:**

In this experimental study, a total of 100 different mammalian cell lines contaminated with different *Mycoplasma* species were evaluated by microbiological culture (as the gold standard method), indirect DNA fluorochrome staining, enzymatic (MycoAlert™), and universal or species-specific polymerase chain reaction (PCR) detection methods. In this study, animal and human cell lines available in National Cell Bank of Iran, were treated with Plasmocure™. The treatment efficacy and cytotoxicity of Plasmocure™ were compared with those of commonly used antibiotics such as BM- cyclin, Plasmocin™, MycoRAZOR™, sparfloxacin and enrofloxacin.

**Results:**

Plasmocure™ is comprised of two antibiotics that act through various mechanisms of action than those in Plasmocin™. Two-week treatment with Plasmocure™ was enough to completely eliminate *Mycoplasma spp.* A moderate toxicity was observed during *Mycoplasma* treatment with plasmocure™; But, after elimination of *Mycoplasma*, cells were fully recovered. *Mycoplasma* infections were eliminated by Plasmocure™, BM-cyclin, Plasmocin™, MycoRAZOR™, sparfloxacin and enrofloxacin. However, the outcome of the treatment process (i.e. the frequency of complete cure, regrowth or cell death) varied among different antibiotics.

**Conclusion:**

The highest number of cured cell lines was achieved by using Plasmocure™ which also had the lowest regrowth rate after a period of four months. As a conclusion; Plasmocure™ might be considered an effective antibiotic to treat *Mycoplasma* infections in mammalian cell cultures especially for precious or vulnerable cells.

## Introduction

*Mycoplasma spp.* contaminations cause a wide range of economical and biotechnical troubles in cell cultures in biological research laboratories as well as biotechnology companies (1, 2). In 1956, *Mycoplasma* was described as one of the most important contaminants of cell cultures (3). Most of the *Mycoplasma* species are known as saprophytic and commensal microbes in eukaryotes (4, 5). They are the smallest and simplest self-replicating bacteria lacking cell wall properties. The cell membrane of *Mycoplasma *is made of triple-layers of cholesterol. Previous studies indicated that 5-87% of cell lines in different cell banks are infected with *Mycoplasma* strains. Among more than 200 species of known mollicutes, 20 of them have been isolated from infected cell cultures. Eight species of *Mycoplasmas* including *M.arginini, M.fermentans, M.orale, M.hyorhinis, M.hominis, M.salivarium, M.pirum* and *Acholeplasmalaidlawii* are responsible for more than 95% of *Mycoplasma*-related cell culture contaminations (6). *Mycoplasma* contaminations can affect the proliferation, the morphology, as well as the metabolic properties of the infected cells. *Mycoplasma* infections may also alter the genome, transcriptome, and proteome properties of the host cells and alter their plasma membrane antigens (1, 4). 

Methods for eliminating *Mycoplasmas* from cell cultures include physical, chemical, immunological, and antibiotic-based approaches. Nevertheless, the methods of *Mycoplasma* elimination should ideally be simple, rapid, efficient, reliable, and inexpensive. They should also have minimal effects on cultured eukaryotic cells (7, 8). Three groups of antibiotics namely, tetracyclines, macrolides and fluoroquinolones, have been shown to be highly effective against *Mycoplasmas* in patients or in cell culture. Since each antibiotic has a specific activity and might not completely eliminate all the *Mycoplasmas* present in a culture, using a combination Plasmocure™ for Elimination of *Mycoplasma* of antibiotics has been frequently implemented (9, 10). The InvivoGen Company has introduced several antibiotics with different mechanisms of action to treat *Mycoplasma*-contaminated cell cultures. In particular, Plasmocin™ (InvivoGen, USA, Cat No. ant-mpt version 16F09-MM) is used to treat cell lines infected by *Mycoplasmas* and related cell wall-less bacteria. Plasmocin™ can also be used as prophylaxis for *Mycoplasma* and other bacterial contaminations. However, some *Mycoplasmas* have been reported to be resistant to Plasmocin™ (8, 11). To eradicate these *Mycoplasmas*, InvivoGen has developed a new anti*Mycoplasma* agent called Plasmocure™ (Alternative *Mycoplasma* Removal Agent, InvivoGen, USA, Cat No. ant-pc version 16F09-MM). Plasmocure™ is comprised of two antibiotics that act through mechanisms different from those of Plasmocin™ . Two-week treatment with Plasmocure™ is enough to completely eradicate *Mycoplasmas* (12). In the present study, we aimed to compare the efficacy and cytotoxicity of Plasmocure™ versus five other available antibiotics namely, Plasmocin™, BM-cyclin (Roche), MycoRAZOR™, sparfloxacin and enrofloxacin. To this end, we evaluated the effectiveness of these antibiotics in elimination of different *Mycoplasma* species contaminating various mammalian cell lines, at National Cell Bank of Iran (NCBI).

## Materials and Methods

### Cell cultures

In this experimental study, 100 different animal and human cell lines available at NCBI were randomly selected (Table S1) (See Supplementary Online Information at www.celljournal.org). All cell lines were analyzed by indirect DNA fluorochrome staining (DAPI, Roche, Germany), mycoplasma enzymatic detection kit (MycoAlert™, Lonza, Switzerland), universal or species- specific polymerase chain reaction (PCR) detection technique and microbiological culture as the reference method. During the experiments, the cells were incubated at 37°C in 88% humidified air containing 5% CO_2_ and cultured in medium including 10-20% fetal bovine serum (FBS, Gibco®-Invitrogen, USA) (13, 14). In addition, specific media were used for growth factors-dependent cell lines. The following reagents and antibiotics were used in this study: 

### Reagents (cell culture media, growth factors, supplements andantibiotics) 

Dulbecco’s modified eagle medium high glucose (DMEM, Gibco®-Invitrogen, USA), Roswell Park Memorial Institute medium 1640 (RPMI 1640, Gibco®- Invitrogen,UK), F12 nutrient mixture (Hams’F12, Gibco®-Invitrogen, USA), McCoy’s 5A medium (ATCC®, USA), eagle’s minimum essential medium (EMEM, ATCC®, USA), Leibovitz’s L-15 medium (ATCC®, USA), earle’s balanced salt solution (EBSS, Gibco®-Invitrogen, USA), horse serum (Gibco®- Invitrogen, NewZealand), Trypsin-EDTA (Gibco®, USA), fischer’s medium (Gibco®-Invitrogen, USA), penicillin/streptomycin (Gibco®-Invitrogen, USA), non-essential amino acid (NEAA, Gibco®-Invitrogen MEM, USA), oxalate, pyruvate, and insulin (OPI, Sigma-Aldrich®, Germany), human insulin (Sis), bovine insulin (Sigma-Aldrich®, Germany), human endothelial cell growth factor (Sigma-Aldrich®, Germany), MEBM/MEGM (mammary epithelial cell growth) (MEGM™, Lonza, Switzerland) medium, fibroblast growth factor-basic from bovine pituitary (bFGF, Sigma-Aldrich®, Germany), 200 mM L-glutamine (Gibco®-Invitrogen, USA), 100 mM sodium pyruvate (Gibco®-Invitrogen, USA), oxalate, sodium bicarbonate (Sigma Aldrich®, Germany), 2-mercaptoethanol (0.05 mM 2ME, Sigma-Aldrich®, Germany), hypoxanthine (Sigma Aldrich®, USA), thymidine (Sigma-Aldrich®, Germany), epidermal growth factor (EGF, Sigma-Aldrich®, Germany), granulocyte macrophage colony-stimulating factor (GM-CSF) recombinant human protein (Gibco®- Invitrogen, USA). At the beginning, culture media, FBS, trypsin and phosphate-buffered saline (PBS, Sigma-Aldrich®, Germany) were analyzed and checked for *Mycoplasma* contamination by above-mentioned methods. For every harvested cell line, *Mycoplasma* contamination was evaluated after 3-5 days of culture in an antibiotic-free medium. In order to confirm the absence of contamination with other microorganisms, cell lines were examined through the quality control of microbiological culture (14, 15). Cells were treated with antibiotics including Plasmocure™ (InvivoGen, USA), BM-cyclin (Roche, Germany), Plasmocin™ (InvivoGen, USA), MycoRAZOR™ (Biontex, Cambio Ltd), sparfloxacin (Zagam®) (Sigma-Aldrich®, Biochemica, Germany) and enrofloxacin (Baytril®) (Sigma-Aldrich®, Biochemica, Germany). The Plasmocure™ cytotoxicity and efficacy for eradication of *Mycoplasma* contamination, as well as the frequency of *Mycoplasma* regrowthwere compared with those of the above-mentioned antibiotics (Table 1).

The working concentrations of Plasmocure™, BMcyclin (Roche), Plasmocin™ and MycoRAZOR™ were chosen according to the manufacturer’s instructions. Furthermore, sparfloxacin and enrofloxacin working concentrations were determined according to previously published reports (7, 12, 16-18). Following treatment with these reagents, the cells were cultured without penicillin, streptomycin or other commonly-used antibiotics (i.e. under antibiotic-free conditions) for at least another 1-2 weeks prior to testing for residual *Mycoplasma* contamination. All the cured cultures were re-examined for regrowth of *Mycoplasmas* for 4 months following the treatment (10).

**Table 1:** Protocols suggested for elimination of Mycoplasma contamination using different antibiotics, including treatment periods and final concentration of each antibiotic


Brand name	Reagent (category)	Mode of action (inhibition of)	Effect on bacteria	Treatment period	Final concentration (μg/ml)

Plasmocure^™^	ND	Protein synthesis	Unpublished	14 days	50
Plasmocin^™^	ND	Protein synthesis, DNA replication	Unpublished	14 days	25
BM-cyclin	I=tiamulin (macrolid)	Protein synthesis	Bacteriostatic	3×3 days	10 (4 µl/ml)
	II=minocycline (tetracycline)	Protein synthesis	Bacteriostatic	3×4 days	5 (4 µl/ml)
MycoRAZOR^™^	Antibiotic mixture in PBS	Protein synthesis	Unpublished	3-5 passes	10 (20 µl/ml)
Zagam^®^	Sparfloxacin (quinolone)	DNA and RNA synthesis	Bactericidal	7 days	10 (1 µl/ml)
Baytril^®^	Enrofloxacin (quinolone)	Nucleic acid synthesis	Bactericidal	7 days	25 (25 µl/ml)


ND; Not defined and PBS; Phosphate-buffered saline.

### Detection of mollicutes

#### Detection of *Mycoplasma* contamination by microbiological culture

The suspended cells (1 ml) were added to 10 ml of Pleuropneumonia-Like Organisms (PPLO) broth medium (BD Difco™, USA) supplemented with 10% horse serum (Gibco®, New Zealand), 1% yeast extract agar (Sigma- Aldrich®, Germany), L-arginine (Sigma-Aldrich®, Germany), D-glucose (Dextrose, Gibco®, USA) and cultured at 37°C for 48-72 hours. In the next step, PPLO medium was vigorously stirred to observe monotonous turbidity. After centrifugation at 1500 rpm for 15 minutes, the precipitate (100 µl) was transferred to a solid PPLO agar (BD Difco™, USA) culture plate and incubated at 37°C for 4-6 weeks. Microscopy observation was used to investigate the formation of non-typical colonies or egg form of *Mycoplasma* colonies, every 3-4 days (1). 

### Detection of *Mycoplasma* contamination by indirect DNA DAPI staining 

This experiment was performed according to previouspublished reports (21, 22). Briefly, cells were cultured oncover slips and stained with 4´, 6-diamidine-2´-phenylindoledihydrochloride (DAPI, Roche, Germany) working solutionin methanol (1 µg/ml) at 37°C for 15 minutes. *Mycoplasma* bodies were detected as polymorphous particles with bluefluorescence. For indirect staining, the supernatants of cell cultures that were suspected to be contaminated with *Mycoplasma*, were added to the *Mycoplasma*-free Vero cell line (NCBI C101b, National Cell Bank of Iran) (19, 20). 

### Detection of *Mycoplasma* contamination by MycoAlert™ *Mycoplasma* detection kit 

The enzymatic MycoAlert™ *Mycoplasma* detection kit was used according to the manufacturer’s instruction. Briefly, the ratio of the ATPs level in each sample before (Reading A) and after (Reading B) the addition of MycoAlert™ substrate, was considered an indicator for the presence of *Mycoplasma* contamination. The presence of contamination was proved if the Reading B/Reading A ratio was greater than 1(1, 21, 22).

### *Mycoplasma* detection using universal and specific polymerase chain reaction method 

**Fig.1:**
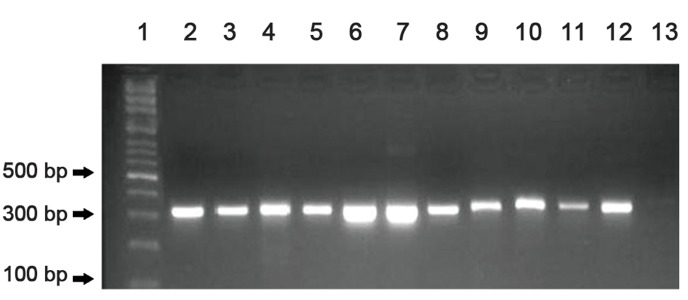
Polymerase chain reaction (PCR) gel electrophoresis of different *Mycoplasma* DNA strains with *Mycoplasma* species-specific primers. Lane 1 DNA size marker (100 bp DNA Ladder, Roche XIV), lane 2 *U.urealyticum* (amplicon size 323 bp), lane 3 *M.fermentans* (amplicon size 324 bp), lane 4 *M.oral* (amplicon size 325 bp), lane 5 *M.salivarium* (amplicon size 324 bp), lane 6 *M.hominis* (amplicon size 301 bp), lane 7 *A.laidlawii* (Amplicon size 300 bp), lane 8 *M.pirum* (Amplicon size 324 bp), lane 9 *M.pneumoniae*(amplicon size 329 bp), lane 10 *M.genitalium* (amplicon size 335 bp), lane 11 *M.hyorhinis* (amplicon size 334 bp), lane 12 *M.arginini* (amplicon size 326 bp), lane 13 DNA-free water (negative control).

The *Mycoplasma* contamination status in 100 cell lines (Table S1) (See Supplementary Online Information at www. celljournal.org) was also determined using PCR-based method as described previously (9, 20). In addition to universal primer pair, 11 species-specific primer pairs were designed based on the 16SrRNA of mollicutes (Fig .1). Sequences of all primers were previously published (20, 21). 

### Determination of *Mycoplasma* contamination status in control cell lines

The control cell lines of the study were assessed for *Mycoplasma* contamination using microbiological culture (as the reference standard test), indirect DNA DAPI staining, enzymatic MycoAlert™ and PCR detection (with universal and specific primers) methods. Vero cell line (NCBI C101a) contaminated with several *Mycoplasma* species, and *Mycoplasma*-free Vero cell line (NCBI C101b) distinct from different sources, were prepared and *Mycoplasma*-free NSO (NCBI C142) cell line were evaluated by above-mentioned methods and confirmed as positive and negative controls, respectively. Three different *Mycoplasma* strains including M.hyorhinis, M.arginini and M.fermentans were detected and identifiedin the positive control cells (Vero cell line contaminated with *Mycoplasma* (NCBI C101a) by species- specific PCR primers) (20, 21).

### Statistical analysis 

Statistical analysis was performed using SPSS 24.0 software (IBM® SPSS® Statistics, USA). Non-parametric Chi-square test (χ^2^) was used for comparisons of two-bytwo in six groups. In Chi-square tests, the difference among the antibiotics for the treatment of *Mycoplasma*-infected cell lines was analyzed and interpreted. Differences with a P<0.05 were considered statistically significant.

## Results

### Characteristics, frequency and treatment of *Mycoplasma* contaminations

In this study, 100 different human and animal cell lines were randomly selected and assessed for *Mycoplasma* contamination. The type of mollicutes in each cell line determined by PCR-based method indicating that 65/100 (65%) of the infected cell cultures was contaminated by one *Mycoplasma* species. Moreover, 19/100 (19%) samples were contaminated with two species and 16/100 (16%) were contaminated with three different species (Table S1) (See Supplementary Online Information at www.celljournal.org). *M.hyorhinis* was detected in 46/100 (46%) of the studied samples, *M.arginini* in 40/100 (40%), *M.fermentans* in 32/100 (32%), *M.orale* in 12/100 (12%), *A.laidlawii* in 6/100 (6%), *M.salivarium* in 4/100 (4%), *M.pirum* in 3/100 (3%), and *M.hominis, M.genitalium, U.urealyticum* and *M.pneumoniae* in 2/100 (2%). 

### Eradication of *Mycoplasma* contaminations

**Fig.2:**
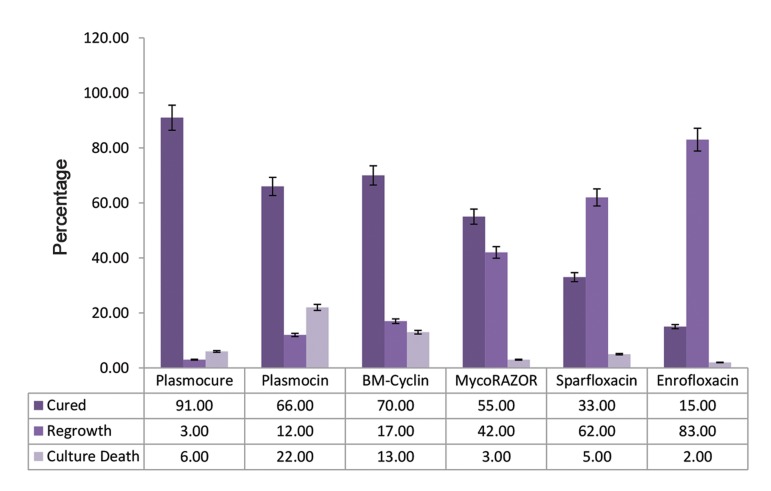
Overall results of the treatment of *Mycoplasma*-positive cell cultures with six antibiotics including Plasmocure™, Plasmocin™, BM-cyclin (Roche), MycoRAZOR™, Sparfloxacin and Enrofloxacin.

The results obtained from *Mycoplasma* treatment process are summarized in Table S1 (See Supplementary Online Information at www.celljournal.org) and Figure 2. *Mycoplasma* infections were eliminated by Plasmocure™, BM-cyclin (Roche), Plasmocin™, MycoRAZOR™,sparfloxacin and enrofloxacin in 91, 70, 66, 55, 33 and 15% of the contaminated cell cultures, respectively. Furthermore, decontamination was confirmed by PCR, as no *Mycoplasma* was detected in cured cell cultures 14 days after the completion of the treatment period. *Mycoplasma* regrowth (reinfection or recurrent infection) was observed in 3, 12, 17, 42, 62 and 83% of the cured cell lines four months after treatment with Plasmocure™, Plasmocin™, BMcyclin (Roche), MycoRAZOR™, sparfloxacin and enrofloxacin, respectively. According to the obtained results, the highest level (22%) of cell cytotoxicity (culture death) was observed among Plasmocin-treated cell lines. While, BM-cyclin (Roche), Plasmocure™, sparfloxacin, MycoRAZOR™andenrofloxacin were cytotoxic to up to 13, 6, 5, 3 and 2% of the studied cell lines, respectively (Table S1 [See Supplementary Online Information at www.celljournal.org], Fig .2). The outcome in the 6 groups of antibiotics showed a significant difference in two-by-two comparison antibiotics in reciprocal case (Table 2). There were significant differences between Plasmocure™and other antibiotics (P=0.001) with regard to treatment of contaminated cell cultures (Table 2, Fig .2). 

However, there was no significant difference between Plasmocin™ and BM-cyclin in the comparison of the treatment outcome (P=0.193). Overall, results reported on antibiotic treatments of cell cultures by different studies are summarized in Table 3. 

**Table 2:** Two-by-two comparison between the antibiotics evaluated in current study with respect to effectiveness in elimination of Mycoplasma


Row	Antibiotic	Number of cured	Number of regrowth	Number of culture death	Antibiotic	P value

1	Plasmocure^™^	91	3	6	Plasmocin^™^	0.001^*^
					BM-cyclin	0.001^*^
					MycoRAZOR^™^	0.001^*^
					Sparfloxacin	0.001^*^
					Enrofloxacin	0.001^*^
2	Plasmocin^™^	66	12	22	BM-cyclin	0.193
					MycoRAZOR^™^	0.001^*^
					Sparfloxacin	0.001^*^
					Enrofloxacin	0.001^*^
3	BM-cyclin	70	17	13	MycoRAZOR^™^	0.001^*^
					Sparfloxacin	0.001^*^
					Enrofloxacin	0.001^*^
4	MycoRAZOR^™^	55	42	3	Sparfloxacin	0.007^*^
					Enrofloxacin	0.001^*^
5	Sparfloxacin	33	62	5	Enrofloxacin	0.004^*^
6	Enrofloxacin	15	83	2	-	-


*; P<0.05.

**Table 3:** The results of different studies reported antibiotic treatment of Mycoplasmas-contaminated cell cultures


Antibiotics	Plasmocure^™^			Plasmocin^™^			BM-cyclin (Roche)			MRA			MycoRAZOR^™^			Ciprofloxacin			Sparfloxacin			Enrofloxacin		
References	C	R	D	C	R	D	C	R	D	C	R	D	C	R	D	C	R	D	C	R	D	C	R	D

Molla Kazemiha et al. (10)	-	-	-	65	10	25	66.25	16.25	17.50	31.25	58.75	10	-	-	-	20	80	0	-	-	-	-	-	-
Molla Kazemiha et al. (9)	-	-	-	-	-	-	100	12.5	17.5	70	62.5	12.5	-	-	-	42.5	82.5	0	-	-	-	-	-	-
Uphoff et al. (12)	-	-	-	84.5	10.3	5.2	86.4	6.8	6.8	-	-	-	-	-	-	-	-	-	-	-	-	73.8	23.8	2.4
Uphoff and Drexler (16)	-	-	-	-	-	-	82	7	11	66	24	10	-	-	-	77	17	6	85	12	3	73	19	8
Fleckenstein and Drexler (35)	-	-	-	-	-	-	84	5	11	64	22	13	-	-	-	77	14	9	-	-	-	-	-	-
Current study	91	3	6	66	12	22	70	17	13	-	-	-	55	42	3	-	-	-	33	62	5	15	83	2


The data present the outcome of the experimentsforthe cell lines treated. The values indicate the frequency of each outcome aspercentage for each antibiotic. MRA; *Mycoplasma* removal agent, C; Cure, R; Regrowth, D; Death of culture, and -; Not tested.

## Discussion

*Mycoplasma* contamination remains one of the major problems in cell culture laboratories. Mycoplasmas can cause significant biological changes in cultured mammalian cells. In fact, consequences of *Mycoplasma* contamination are unpredictable and may affect molecular and cellular properties of the infected cells (7, 23, 24). In particular, *Mycoplasma* contamination can lead to attenuation of cell proliferation, unreliable experimental results, and potentially unsafe biological products (1, 25). *Mycoplasmas* are resistant to many antibiotics which are commonly used in cell culture. This problem has become more widespread since the introduction of more sensitive, rapid, and efficient methods of detection of *Mycoplasmas* in cell culture. Recent reports have estimated that *Mycoplasma* contamination may affect up to 83% of cell cultures worldwide (2, 4, 6, 10). Administration of antibiotics is the most reliable and efficient approach to combat *Mycoplasma* contamination. However, it is important to determine the efficacy and potential side-effects of the antibiotics on the eukaryotic cells in culture. For treatment of irreplaceable, valuable and expensive cell lines, the safety of the antibiotics used against *Mycoplasma* contamination, is particularly important (2, 4, 8). In addition, some cell types may be infected with different *Mycoplasma* species making it difficult to draw an accurate conclusion on choosing an antibiotic (7, 26, 27). In our experience, Plasmocure™ was able to cure 91 out of 100 cell lines (91%), with 3 cases of regrowth (3%) and 6 of cell death (6%). Plasmocure™ is comprised of two bactericidal components belonging to different antibiotic families. They both act by inhibiting protein synthesis but through distinct mechanisms. One of these antibiotic binds to the 50s subunit of the bacterial ribosomeand blocks the peptidyltransferase activity. The other antibiotic which binds to isoleucyl-tRNAsynthetase prevents the addition of isoleucine to bacterial proteins (28-30). 

Remarkably, the problem of regrowth in Plasmocure™treated cell lines was resolved by using Plasmocin™or BMcyclin (Roche), and vice versa. In case of cytotoxicity and cell death, especially in severe and intensive contaminations with multiple *Mycoplasma* strains, BM-cyclin (Roche) and Plasmocin™ were used successfully. In case of mild contaminations, particularly for vulnerable or precious cells such as myeloma, lymphoma, hybridoma or primary cultures, MycoRAZOR™ along with fluoroquinolones (sparfloxacin and enrofloxacin) can be used as alternative antibiotics. Plasmocin™ (comprised of a macrolide and a quinolone) acts on the protein machinery and DNA replication by interfering with ribosomal translation and replication fork, respectively. BM-cyclin (Roche) binds to the 30S and 50S ribosomal subunits and inhibits protein synthesis. According to the manufacturer’s information, the bacteriostatic components of BM-cyclin (Roche) are pleuromutilin and tetracycline whereas Plasmocin™ is composed of a macrolide and a quinolone (10-12, 31, 32). MycoRAZOR™ is an effective antibiotic against *Mycoplasma*, which is active at low concentrations against various *Mycoplasma* species. It diminishes *Mycoplasmas* protein biosynthesis by interfering with their ribosome function as well as DNA transcription. MycoRAZOR™ has no undesired impact on the eukaryotic cells in the culture. On the other hand, sparfloxacin and enrofloxacin as members of the fluoroquinolone family, inhibit bacterial DNA gyrase and DNA replication (33).

Zakharova et al. (32) showed that Plasmocin™ can effectively treat chronic *Mycoplasma *infections. Similarly, Molla Kazemiha et al. (10) observed that *Mycoplasma* infections were eradicated by Plasmocin™, BM-cyclin (Roche), ciprofloxacin and MRA (Mycoplama Removal Agent, AbDSerotec, UK), in 65, 66.25, 20 and 31.25% of the cell lines, respectively. In addition, cytotoxicity was reported in 0, 10, 17.5 and 25% of the cell lines treated with ciprofloxacin, MRA, BM-cyclin (Roche) and Plasmocin™, respectively. Nevertheless, recurrent *Mycoplasmas* infection was observed in 10 to 80% of the studied cell lines after four months. In another study done by Molla Kazemiha et al. (9), *Mycoplasma* infections were eradicated in 100, 70 and 42% of the infected cell lines treated with BM-cyclin (Roche), MRA and ciprofloxacin, respectively. It is noteworthy that, the risk of cell culture loss was 0, 12.5 and 17.5% for ciprofloxacin, MRA and BM-cyclin (Roche), respectively. However, 82.5 (for ciprofloxacin), 62.5 (for MRA) and 12% (BM-cyclin) of the treated cell lines showed *Mycoplasma* regrowth (9, 18).

In this study, we observed high frequency of *Mycoplasma* resistance/regrowth following treatment with enrofloxacin (83%), sparfloxacin (62%) or MycoRAZOR™ (42%). Plasmocure™, BM-cyclin (Roche) and Plasmocin™ were effective especially in elimination of *M.hyorhinis, M.arginini, M.fermentans* and *M.orale* which were resistant to the other antibiotics used in this study. Plasmocure™ showed the lowest frequency (3%) of regrowth in our experiments while regrowth was observed in 17 and 12% of cell lines treated with BM-cyclin (Roche) and Plasmocin™, respectively. Plasmocure™, BM-cyclin (Roche), Plasmocin™ and MycoRAZOR™ effectively eradicated mollicutes and cured 91, 70, 66 and 55% of the cell lines, respectively. However, sparfloxacin and enrofloxacin were considerably less efficient as they cured only 33 and 15% of the cell lines, respectively. 

Plasmocin™ caused the highest rate of culture death (22%), although, it targets the prokaryotic DNA replication and protein synthesis machineries which are different from those of eukaryotic cells. In addition, MycoRAZOR™ showed lower cytotoxicity on the studied cell lines (culture death of 3%), which might reduce the risk of culture loss. Therefore, it may be recommended as the first-line alternative especially in case of expensive or hard-to-obtain cell lines (9, 10, 34). Moreover, quinolones and fluoroquinolones such as ciprofloxacin, enrofloxacin or sparfloxacin were used along with MycoRAZOR™ without increased cytotoxicity (35).

Finally, we observed that the combination of two or more antibiotic with different mechanisms of action makes the interpretation of the results more complicated. Based on our experiments, this might increase the risk of culture death or antibiotic resistance of *Mycoplasmas*. Thus, we suggest using two or more antibiotics in alternating periods for a successful treatment or eradication of *Mycoplasma* contamination. For example, BM-cyclin (Roche) or MycoRAZOR™ (inhibitors of protein synthesis) can be used alternately along with ciprofoxacin, enrofloxacin or sparfloxacin (inhibitors of DNA gyrase activity and DNA replication) with specified intervals during the treatment period. 

## Conclusion

This report suggests Plasmocure™ as a reliable anti- *Mycoplasma* agent in comparison with other antibiotics for elimination of *Mycoplasma* contamination in cultured cells. As a conclusion, we recommend Plasmocure™ as an effective antibiotic for the treatment of *Mycoplasma* infections in mammalian cell cultures especially for precious or vulnerable cells. These findings may also help researches at biotechnology laboratories for selection of appropriate antibiotics for treatment of *Mycoplasma* contamination in cell cultures. 

## Supplementary PDF


