# Supplementary material for: Effectiveness of Plasmocure™ in Elimination of Mycoplasma Species from Contaminated Cell Cultures: A Comparative Study versus Other Antibiotics
Source: Cell J. 2019 Feb 25;21(2):143–9. doi: 10.22074/cellj.2019.5996 (PMC6397598; doi:10.22074/cellj.2019.5996)
Supplement: Supplementary file 1 [file Cell-J-21-143-s01.pdf]

CELL JOURNAL (Yakhteh)

Supplementary Information for

Effectiveness of Plasmocure™ in Elimination of *Mycoplasma* Species from Contaminated Cell Cultures: A Comparative Study versus Other Antibiotics

Vahid Molla Kazemiha, M.Sc.[1], Shahram Azari, Ph.D.[1], Mahdi Habibi-Anbouhi, Ph.D.[1], Amir Amanzadeh, Ph.D.[1], Shahin Bonakdar, Ph.D.[1], Mohammad Ali Shokrgozar, Ph.D.[1]*, Reza Mahdian, M.D., Ph.D.[2]*

1. National Cell Bank of Iran, Pasteur Institute of Iran, Tehran, Iran
2. Department of Molecular Medicine, Pasteur Institute of Iran, Tehran, Iran

**Corresponding Addresses: P.O.Box: 1316943551, National Cell Bank of Iran, Pasteur Institute of Iran, Tehran, Iran*
P.O.Box: 1316943551, Department of Molecular Medicine, Pasteur Institute of Iran, Tehran, Iran
Emails: mashokrgozar@pasteur.ac.ir, dr.reza.mahdian@gmail.com

Table S1: Specific polymerase chain reaction (PCR) analysis for *Mycoplasma* detection and antibiotic treatment in the 100 cell lines

No*	Cell line name	Cell type	NCBI Code	Plasmoc	Pla	BM	MycoR	Spar	Enr	Effective antibiotics	*Mycoplasma* Species
1	MCF-7	Human breast adeno carcinoma	C135	O	O	O	O	O	Δ	Plasmoc, Pla, BM MycoR , Spar	*Mhy*
2	T-47D	Human breast ductal carcinoma	C203	O	O	O	O	Δ	Δ	Plasmoc, Pla, BM, MycoR	*Mfe*
3	RAJI	Human burkitt's lymphoma	C127	O	X	X	O	O	Δ	Plasmoc, MycoR, Spar	*Mfe*
4	MDA-MB231	Human breast adenocarcinoma	C578	O	O	O	O	Δ	Δ	Plasmoc, Pla, BM, MycoR	*Mar*
5	HEP G2	Human hepatocyte carcinoma	C158	O	O	O	O	X	Δ	Plasmoc, Pla, BM, MycoR	*Mhy*
6	Caco2	Human colon adenocarcinoma	C139	O	O	Δ	Δ	Δ	Δ	Plasmoc, Pla	*Mar, Mhy*
7	HFFF-PI6	Human fetal foreskin fibroblast	C170	O	X	O	O	Δ	Δ	Plasmoc, BM, MycoR	*Mor*
8	Hep2	Human larynx carcinoma	C144	O	O	O	Δ	Δ	Δ	Plasmoc, Pla, BM	*Mhy*
9	MRC-5	Human foetal lung fibroblast	C125	X	X	O	O	O	O	BM, MycoR, Spar, Enr	*Mar*
10	Saos-2	Human osteogenic sarcoma	C453	O	O	Δ	Δ	Δ	Δ	Plasmoc, Pla	*Mar, Mfe, Mhy*
11	P3X63Ag8.653	Mouse-myeloma	C109	O	X	X	O	Δ	Δ	Plasmoc, MycoR	*Mar*
12	Vero	Monkey kidney	C101	O	O	O	Δ	Δ	Δ	Plasmoc, Pla, BM	*Mar, Mfe, Mhy*
13	SP2/0-Ag14	Hybridoma mouse myeloma	C129	O	Δ	O	Δ	Δ	Δ	Plasmoc, BM	*Mfe, Mhy*
14	McCoy	Mouse synovial tissue fibroblast	C123	O	O	O	O	Δ	Δ	Plasmoc, Pla, BM, MycoR	*Mar*
15	PC3	Human prostate adenocarcinoma	C427	O	O	O	Δ	Δ	Δ	Plasmoc, Pla ,BM	*Mhy*
16	J774A.1	Mouse monocyte/ macrophage	C483	O	Δ	O	O	X	Δ	Plasmoc, BM, MycoR	*Mar, Mhy*
17	DAUDI	Human burkitt's lymphoma	C112	O	X	O	O	Δ	Δ	Plasmoc, BM, MycoR	*Mor*
18	THP-1	Human acute Monocytic leukemia	C563	O	O	O	O	Δ	Δ	Plasmoc, Pla, BM, MycoR	*Mor*
19	CCRF-CEM	Human acute lymphoblasttic leukemia	C105	O	X	O	O	O	O	Plasmoc, BM, MycoR, Spar, Enr	*Mfe*
20	PANC-1	Human pancreas duct epithelial carcinoma	C556	O	O	O	Δ	Δ	Δ	Plasmoc, Pla, BM	*Mar, Mfe, Mhy*
21	BT-474	Human breast ductal carcinoma	C435	O	O	O	O	O	Δ	Plasmoc, Pla, BM MycoR, Spar	*Mar*

Table S1: Continued

No*	Cell line name	Cell type	NCBI Code	Plasmoc	Pla	BM	MycoR	Spar	Enr	Effective antibiotics	*Mycoplasma* Species
22	STO	Mouse SIM fetal fibroblast	C537	O	Δ	O	Δ	Δ	Δ	Plasmoc, BM	*Mhy*
23	SKBR3	Human breast adenocarcinoma	C207	Δ	O	Δ	Δ	Δ	Δ	Pla	*Mor, Mhy*
24	Hek293	Human embryonic kidney cells	C497	O	Δ	O	Δ	Δ	Δ	Plasmoc, BM	*Mhy*
25	PC12 (Suspension)	Rat adrenal pheochromocytoma	C189	O	X	O	O	O	Δ	Plasmoc, BM, MycoR, Spar	*Mhy*
26	WEHI-164	Mouse BALB/c fibrosarcoma	C200	O	O	O	O	O	Δ	Plasmoc, Pla, BM, MycoR, Spar	*Mhy*
27	K562	Human CML	C122	O	O	O	O	Δ	Δ	Plasmoc, Pla, BM, MycoR	*Ala*
28	SK-N-MC	Human neuroblastoma	C535	O	O	Δ	Δ	Δ	Δ	Plasmoc, Pla	*Mfe*
29	HL60	Human promyelocytic leukemia	C217	O	O	O	O	Δ	Δ	Plasmoc, Pla, BM, MycoR	*Mar, Msa*
30	U937	Human histiocytic lymphoma	C130	O	O	O	O	Δ	Δ	Plasmoc, Pla, BM, MycoR	*Mfe*
31	F3B6	Human × mouse heterohybridoma	C197	X	X	X	O	O	Δ	MycoR, Spar	*Mar, Mor*
32	CoR-L-105	Human lung adenocarcinoma	C113	O	O	O	Δ	Δ	Δ	Plasmoc, Pla, BM	*Mor*
33	HGF3-PI 53	Human gingival fibroblast	C502	O	X	O	O	Δ	Δ	Plasmoc, BM, MycoR	*Mhy*
34	MDA-MB-468	Human breast adenocarcinoma	C208	O	O	Δ	Δ	Δ	Δ	Plasmoc, Pla	*Mar, Mfe, Mhy*
35	$A2780_S$	Human ovarian carcinoma (sensitive to cisplatin)	C461	O	O	X	O	O	Δ	Plasmoc, Pla, MycoR, Spar	*Mhy*
36	MG63	Human osteosarcoma	C555	O	O	Δ	Δ	Δ	Δ	Plasmoc, Pla	*Mar*
37	L929	Mouse connective tissue fibroblast	C161	O	O	O	O	Δ	Δ	Plasmoc, Pla, BM, MycoR	*Mfe*
38	CT26	Mouse colon carcinoma	C532	O	Δ	O	Δ	Δ	Δ	Plasmoc, BM	*Mhy*
39	Hela	Human cervix carcinoma	C115	O	O	Δ	Δ	Δ	Δ	Plasmoc, Pla	*Mar, Mfe, Mhy*
40	NIH3T3	Mouse swiss embryo fibroblast	C156	O	O	O	O	O	Δ	Plasmoc, Pla, BM, MycoR, Spar	*Mhy*
41	EL4	Mouse T cell lymphoma	C114	O	X	O	Δ	Δ	Δ	Plasmoc, BM	*Mhy*
42	A-431	Human squamous carcinoma	C204	O	O	Δ	Δ	Δ	Δ	Plasmoc, Pla	*Mar, Mhy*

Table S1: Continued

No[*]	Cell line name	Cell type	NCBI Code	Plasmoc	Pla	BM	MycoR	Spar	Enr	Effective antibiotics	*Mycoplasma* Species
43	B95-8	Marmoset EBV transformed lymphocytes	C110	O	X	O	Δ	Δ	Δ	Plasmoc, BM	*Mar, Mfe*
44	CHO	Chinese hamster ovary	C111	O	O	O	O	O	O	Plasmoc, Pla, BM, MycoR, Spar, Enr	*Mar*
45	JIYOYE	Human burkitt's lymphoma	C117	O	O	X	O	O	Δ	Plasmoc, Pla, MycoR, Spar	*Mor*
46	HUT-78	Human cutaneus T cell lymphoma	C185	O	O	O	Δ	Δ	Δ	Plasmoc, Pla, BM	*Mar, Mfe, Ala*
47	LNCap-FGC-10	Human prostate cancer	C439	O	O	O	O	Δ	Δ	Plasmoc, Pla, MycoR, Spar	*Mhy*
48	BCL1 clone 5B1b	Mouse lymphoma	C551	X	X	X	O	O	O	MycoR, Spar, Enr	*Ala*
49	BW5147	Mouse thymoma	C542	O	X	O	O	X	Δ	Plasmoc, BM, MycoR	*Mhy*
50	PC12	Rat adrenal fibroblast pheochromocytoma	C153	O	Δ	O	Δ	Δ	Δ	Plasmoc, BM	*Mhy*
51	Seraphina	Human burkitt's lymphoma	C102	O	O	O	O	O	O	Plasmoc, Pla, BM, MycoR, Spar, Enr	*Mfe*
52	LCL-PI 12	Human EBV transformed cord blood B cell	C178	O	O	O	O	O	O	Plasmoc, Pla, BM, MycoR, Spar, Enr	*Mfe*
53	HSF-PI 17	Human skin fibroblast	C193	O	X	O	O	X	Δ	Plasmoc, BM, MycoR	*Mfe*
54	B65	Rat nerrous tissue neuronal	C134	O	X	X	O	O	Δ	Plasmoc, MycoR, Spar	*Mar*
55	Peer	Human acute T cell lymphoblastic leukemia	C511	O	X	O	O	X	X	Plasmoc, BM, MycoR	*Ala*
56	HL60 / mix 1	Human acute promyelocytic leukemia	C553	O	X	O	O	Δ	Δ	Plasmoc, BM, MycoR	*Mar, Mpi*
57	HPA / ALL	Human T cell acute lymphoblastic leukemia	C213	X	X	X	X	O	O	Spar, Enr	*Mfe*
58	LB3.1 (HB298)	Mouse anti human HLA-DR alpha chain	H195	O	O	Δ	Δ	Δ	Δ	Plasmoc, Pla	*Mar, Mhy, Mho*
59	A3.6B10 (HB12318)	Mouse anti human CTLA-4 (CD152)	H196	O	O	Δ	Δ	Δ	Δ	Plasmoc, Pla	*Mar, Mhy, Mho*
60	Nalm6	Pre B cell leukemia	C212	O	O	O	O	O	O	Plasmoc, Pla, BM, MycoR, Spar, Enr	*Mfe*
61	KG1	Human caucasian bone marrow myeloid leukemia	C119	O	O	O	O	O	O	Plasmoc, Pla, BM, MycoR, Spar, Enr	*Msa*
62	CHO DG-44	Dihydrofolate reductase-deficient CHO	C576	O	O	O	O	O	O	Plasmoc, Pla, BM, MycoR, Spar, Enr	*Mar*
63	G28	Anti human CD40	H150	O	Δ	X	Δ	O	Δ	Plasmoc, Spar	*Mar, Mhy, Msa*

Table S1: Continued

No*	Cell line name	Cell type	NCBI Code	Plasmoc	Pla	BM	MycoR	Spar	Enr	Effective antibiotics	*Mycoplasma* Species
64	NS1	Mouse myeloma	C522	O	O	O	O	O	O	Plasmoc, Pla, BM, MycoR, Spar, Enr	*Mhy*
65	MDBK	Bovine normal kidney epithelial cells	C500	O	O	X	Δ	Δ	Δ	Plasmoc, Pla	*Mar, Mhy*
66	Rael	Human burkitt,s lymphoma	C186	X	O	X	Δ	Δ	Δ	Pla	*Mor*
67	DFW	Human melanoma	C496	O	O	O	O	O	O	Plasmoc, Pla, BM, MycoR, Spar, Enr	*Mfe*
68	LCL PI7	Human EBV-transformed peripheral blood B cells	C175	O	O	O	O	O	Δ	Plasmoc, Pla, BM, MycoR, Spar	*Mfe*
69	B16F10	Mouse melanoma	C540	O	O	Δ	Δ	Δ	Δ	Plasmoc, Pla	*Mar, Mfe, Mhy*
70	ASPC1	Human pancrease adenocarcinoma	C558	O	O	Δ	Δ	Δ	Δ	Plasmoc, Pla	*Mhy, Mpi*
71	HT29	Human colon adenocarcinoma	C466	O	O	O	Δ	Δ	Δ	Plasmoc, Pla, BM	*Mhy*
72	LS180	Human colon adenocarcinoma	C508	O	X	O	O	O	Δ	Plasmoc, BM, MycoR, Spar	*Mar*
73	MOLT-17	Human T cell leukemia	C516	O	O	O	O	O	O	Plasmoc, Pla, BM, MycoR, Spar, Enr	*Mfe*
74	CAOV-4	Human ovary adenocarcinoma	C595	O	O	O	Δ	Δ	Δ	Plasmoc, Pla, BM	*Mfe, Mge*
75	T45	Human T cell acute lymphoblastic leukemia	C180	X	X	O	O	O	Δ	BM, MycoR, Spar	*Mpi*
76	DND-41	Human T cell acute lymphoblastic leukemia	C183	O	O	O	O	O	Δ	Plasmoc, Pla, BM, MycoR, Spar	*Mor*
77	RpmI8402	Human T cell acute lymphoblastic leukemia	C184	O	O	X	X	O	O	Plasmoc, Pla, Spar, Enr	*Mfe*
78	LL/2(LLC1)	Mouse lewis lung carcinoma	C587	O	O	Δ	Δ	Δ	Δ	Plasmoc, Pla	*Mar, Mhy, Ala*
79	BHK21-2PCLone13	Human syrian kidney (suspension culture)	C108	O	Δ	O	Δ	Δ	Δ	Plasmoc, BM	*Mar, Mfe*
80	HFIF PI4	Human fetal liver fibroblast	C168	O	X	O	O	Δ	Δ	Plasmoc, BM, MycoR	*Mfe, Mhy, Uur*
81	DU145	Human prostatic carcinoma	C428	O	O	O	Δ	Δ	Δ	Plasmoc, Pla, BM	*Mar, Mhy, Mpn*
82	SK-MEL-37	Human malignant melanoma	C653	O	O	O	O	Δ	Δ	Plasmoc, Pla, BM, MycoR	*Mar, Mhy*
83	JIMT-1	Human breast carcinoma	C652	O	O	O	Δ	Δ	Δ	Plasmoc, Pla, BM	*Mfe, Mhy*
84	Mv1Lu	American mink-Lung	C647	O	O	Δ	Δ	Δ	Δ	Plasmoc, Pla	*Mar, Mpn*

Table S1: Continued

No[*]	Cell line name	Cell type	NCBI Code	Plasmoc	Pla	BM	MycoR	Spar	Enr	Effective antibiotics	*Mycoplasma* Species
85	CHO-K1	Chinese Hamster Ovary	C645	O	O	O	O	O	Δ	Plasmoc, Pla, BM, MycoR, Spar	*Mar*
86	MCF10A	Human non-tumorigenic breast epithelial	C609	Δ	O	Δ	Δ	Δ	Δ	Pla	*Mfe, Mor, Uur*
87	KATO III	Human gastric carcinoma	C640	O	O	X	X	O	X	Plasmoc, Pla, Spar	*Mar*
88	KPL-1	Human Breast Carcinoma	C643	O	X	O	O	O	Δ	Plasmoc, BM, MycoR, Spar	*Mhy*
89	RAW264.7	Mouse monocyte macrophage	C639	O	Δ	O	Δ	Δ	Δ	Plasmoc, BM	*Mar, Mhy*
90	SW872	Human connective tissue fibrosarcoma	C638	O	O	O	O	Δ	Δ	Plasmoc, Pla, BM, MycoR	*Mor*
91	NFS-60	Mouse myeloblast	C603	O	O	O	O	O	O	Plasmoc, Pla, BM, MycoR, Spar, Enr	*Mfe*
92	4T1	Mouse mammary gland tumor	C604	O	Δ	Δ	Δ	Δ	Δ	Plasmoc	*Mar*
93	BFA	Bovine aorta endothelium foetal	C608	O	O	Δ	Δ	Δ	Δ	Plasmoc, Pla	*Mar, Ala*
94	P3U1	Mouse myeloma	C579	O	O	O	O	Δ	Δ	Plasmoc, Pla, BM, MycoR	*Mhy*
95	C6	Mouse glioma	C575	O	O	O	O	Δ	Δ	Plasmoc, Pla, BM, MycoR	*Mfe*
96	BHY	Human oral squamous carcinoma	C622	Δ	Δ	O	Δ	Δ	Δ	BM	*Mor, Mhy, Msa*
97	U266B1	Human Myeloma	C151	O	O	O	O	Δ	Δ	Plasmoc, Pla, BM, MycoR	*Mfe*
99	OVCAR-3	Human ovary adenocarcinoma	C430	O	Δ	O	Δ	Δ	Δ	Plasmoc, BM	*Mar, Mhy*
100	OLN-93	Rat oligodendroglia	C617	O	O	O	O	Δ	Δ	Plasmoc, Pla, BM, MycoR	*Mhy*

O; Cured, Δ; Regrowth, X; Culture death, Plasmoc; Plasmocure™, Pla; Plasmocin™ , BM; BM-cyclin (Roche), MycoR; MycoRAZOR™, Spar; Sparfloxacin, Enr; Enrofloxacin, NCBI; National Cell Bank of Iran, *Mar; M.arginini, Mfe; M.fermentans, Mor; M.orale, Mhy; M.hyorhinis, Ala; A.laidlawii, Msa; M.salivarium, Mpi; M.pirum, Mho; M.hominis, Mge; M.genitalium, Uur; U.urealyticum, and Mpn; M.pneumoniae.*
